# Double entrapment neuropathy of the ulnar nerve at the elbow and the wrist : double crush syndrome?

**DOI:** 10.1186/s12891-024-07574-z

**Published:** 2024-06-13

**Authors:** Dong Hee Kim, Sung Jin Shin, Jun Yong Park, Sang Hyun Lee

**Affiliations:** 1 Departments of Orthopedic Surgery, Samsung Changwon Hospital, Sungkyunkwan University School of Medicine, Changwon, Republic of Korea; 2 Department of Orthopaedic Surgery, Medical Research Institute, Pusan National University Hospital, Pusan National University School of Medicine, 179, Gudeok-ro seo-gu, Busan, 49241 Republic of Korea

**Keywords:** Cubital tunnel syndrome, Double crush syndrome, Nerve entrapment, Neuropathy, Ulnar nerve

## Abstract

**Background:**

Double crush syndrome refers to a nerve in the proximal region being compressed, affecting its proximal segment. Instances of this syndrome involving ulnar and cubital canals during ulnar neuropathy are rare. Diagnosis solely through clinical examination is challenging. Although electromyography (EMG) and nerve conduction studies (NCS) can confirm neuropathy, they do not incorporate inching tests at the wrist, hindering diagnosis confirmation. We recently encountered eight cases of suspected double compression of ulnar nerve, reporting these cases along with a literature review.

**Methods:**

The study included 5 males and 2 females, averaging 45.6 years old. Among them, 4 had trauma history, and preoperative McGowan stages varied. Ulnar neuropathy was confirmed in 7 cases at both cubital and ulnar canal locations. Surgery was performed for 4 cases, while conservative treatment continued for 3 cases.

**Results:**

In 4 cases with wrist involvement, 2 showed ulnar nerve compression by a fibrous band, and 1 had nodular hyperplasia. Another case displayed ulnar nerve swelling with muscle covering. Among the 4 surgery cases, 2 improved from preoperative McGowan stage IIB to postoperative stage 0, with significant improvement in subjective satisfaction. The remaining 2 cases improved from stage IIB to IIA, respectively, with moderate improvement in subjective satisfaction. In the 3 cases receiving conservative treatment, satisfaction was significant in 1 case and moderate in 2 cases. Overall, there was improvement in hand function across all 7 cases.

**Conclusion:**

Typical outpatient examinations make it difficult to clearly differentiate the two sites, and EMG tests may not confirm diagnosis. Therefore, if a surgeon lacks suspicion of this condition, diagnosis becomes even more challenging. In cases with less than expected postoperative improvement in clinical symptoms of cubital tunnel syndrome, consideration of double crush syndrome is warranted. Additional tests and detailed EMG tests, including inching tests at the wrist, may be necessary. We aim to raise awareness double crush syndrome with ulnar nerve, reporting a total of 7 cases to support this concept.

## Introduction

The concept of double crush syndrome(DCS), first described by Upton and McComas in 1973, posits compression of a specific nerve in the proximal region of the human body can induce vulnerability of the proximal segment of the nerve to secondary compression [1]. In clinical practice, it is not uncommon to observe the coexistence of cervical radiculopathy and carpal tunnel syndrome, which can be regarded as an example of DCS [2]. Hurst et al. have proposed a correlation between cervical arthritis and carpal tunnel syndrome through case reports [3], while Baba reported a correlation with cervical radiculopathy in a retrospective study of patients with carpal tunnel syndrome [4]. Furthermore, it has been confirmed in several studies that the incidence of carpal tunnel syndrome is high in patients diagnosed with cervical radiculopathy [2]. 

In the past, instances of DCS involving the ulnar nerve have been reported among cyclists presenting with concurrent cervical radiculopathy and cubital tunnel syndrome(CuTS) [5]. The occurrence of DCS, characterized by nerve entrapment findings in both the cubital and Guyon`s canals associated with ulnar neuropathy, is exceedingly rare [6–8]. If the two conditions occur simultaneously, it is challenging to differentiate them through clinical physical examination. Although electromyography (EMG) and nerve conduction studies (NCS), which serve as standard diagnostic tests for neuropathy, can be administered, traditional methodologies do not incorporate inching tests at the wrist location, hindering identification of double crush syndrome. In the absence of clinical suspicion by the physician, a proper diagnosis may not be achieved.

We recently encountered seven cases of suspected double compression of the ulnar nerve at both the elbow and wrist. Given the rarity of these patients, finding appropriate guidelines can be challenging. Questions arise about whether to operate on one or both sides in DCS, and whether conservative treatment can be effective. This report details the experiences of eight patients who underwent both surgical and conservative treatments. It describes the features of the EMG/NCS test used for diagnosis and discusses the treatment methods and outcomes.

## Methods

This study was approved by the Institutional Review Board of Samsung Changwon hospital of Sungkyunkwan University. All procedures were performed in accordance with the relevant guidelines and regulations. From 2020 to 2023, a retrospective study was conducted of patients treated at our institution, comprising 7 cases with cubital and ulnar canal ulnar. These patients consisted of 5 males and 2 females, with an average age of 45.6 years (range: 13–66 years), and an average duration of clinical symptoms of approximately 19.4 months (range: 1-120 months). Upon presentation, physical examination revealed complaints of numbness and sensory reduction in the 4th and 5th digits in 5 patients, while digit sensation was normal in 2 patients. Prior to treatment, physical nerve examination confirmed positive Watenberg’s sign, positive Egawa sign, and 1st web space atrophy observed in 5 cases. In digit function evaluation, 1 case demonstrated inability to perform tasks such as tying shoelaces, using chopsticks, and buttoning clothes, while 5 cases reported difficulty but were able to perform these tasks. At presentation, 2 had a history of elbow or wrist trauma, and 1 had chronic kidney disease as a specific underlying condition. Preoperative radiographs revealed no specific findings in the elbow and wrist for all patients. The preoperative McGowan stage was classified as stage stage IIB in 4 cases, stage IIA in 1 case, and stage I in 2 cases.

All patients underwent imaging studies, EMG and NCS. Ulnar neuropathy was confirmed at both the cubital and ulnar canal locations in 7 cases. Especially, an inching test was performed at the wrist in 7 patients. Six patients showed decreased conduction velocity at the wrist. One patient revealed significantly decreased ampilitude findings in the abductor digiti minimi (ADM) muscle or at the first dorsal interosseous (FDI) muscle. Compared to the Flexor carpi ulnaris(FCU) and Flexor digitorum profundus(FDP) muscle, the resting potential of the FCU and FDP muscle is preserved

**Fig. 1:**
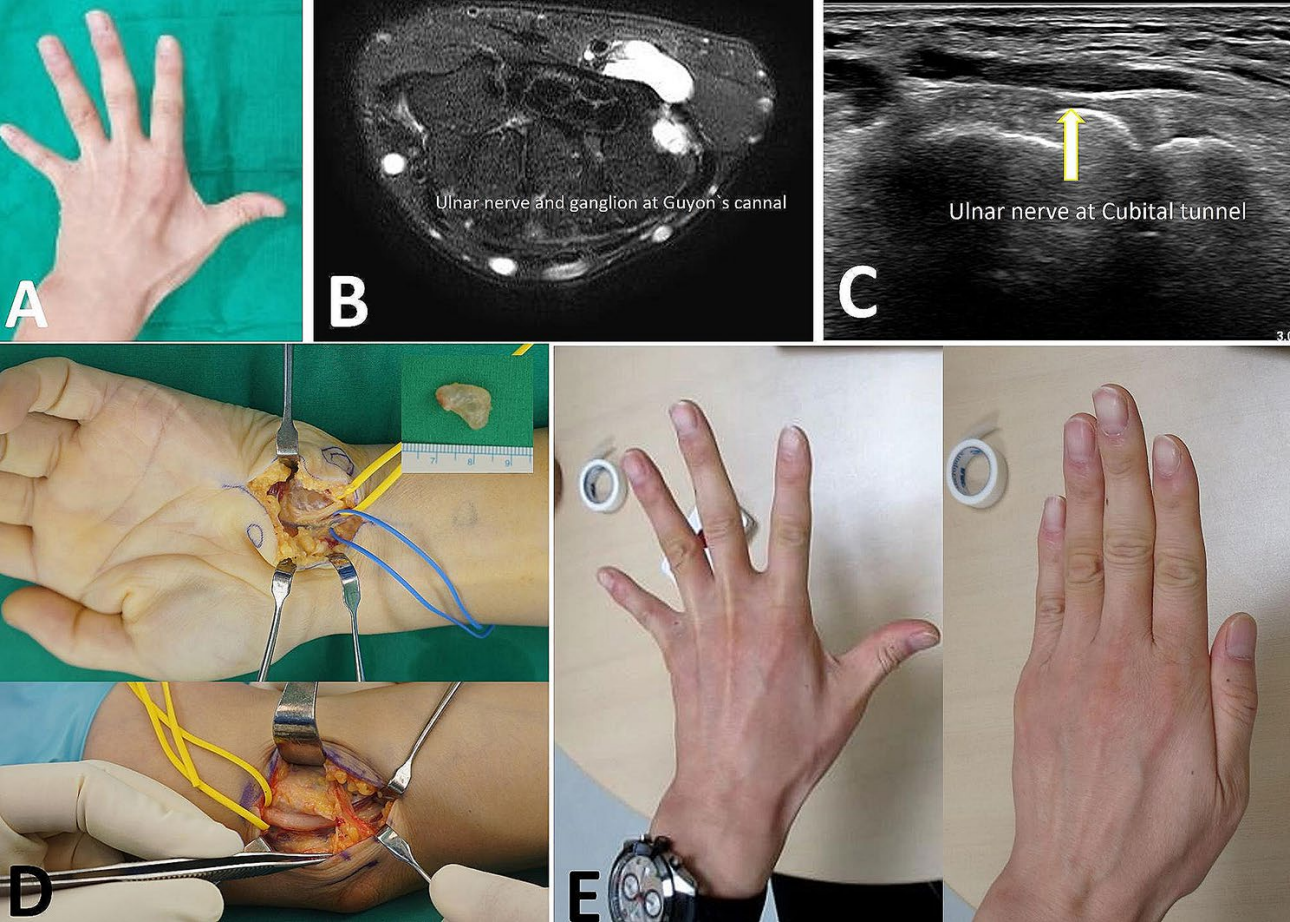
A forty-seven years old male came for left 2nd finger weakness after golf. Double crush syndrome was confirmed by EMG/NCS with Cubital tunnel syndrome (on left elbow) and Guyon’s canal syndrome (on left wrist). (**A**) On physical examination, 1st web space atrophy was observed., (**B**) The preoperative T2 magnetic resonance images of left wrist show a 1.5 cm sized ganglion mass in Guyon’s canal of hypothenar muscle layer. (**C**) The preoperative ultrasonography of left wrist view shows ulnar nerve swelling and a cystic lesion in Guyon’s canal (white arrow). (**D**) Intraoperative exploration, the ulnar nerve was compressed by huge ganglion at left wrist and below left elbow medial epicondyle, the ulnar nerve was compressed with swelling. (**E**) On physical examination of last follow up, 1st web space atrophy was improved. (Mcgowan stage 0)

Preoperative ultrasound examination was conducted in all 4 surgical cases, demonstrating ulnar nerve swelling and decreased echo shadow at the elbow in all cases. Additionally, 2 patients exhibited a thicker deep branch of the ulnar nerve at the wrist compared to the contralateral side. One patient presented with a 3 × 0.6 × 2 cm cystic lesion in the ulnar canal at the wrist compressing the ulnar nerve, confirmed by additional MRI examination Fig. 1. In 1 patient, a tubular hypoechoic lesion measuring 0.1 × 0.2 cm was identified within the ulnar canal at the wrist, extending from the hamate hook level to the deep aspect of the flexor digit minimi (FDM) muscle.

Surgical treatment was administered to 4 cases with persistent or worsening symptoms despite conservative management and McGowan stage IIB or higher, while conservative treatment was maintained for 3 cases. Four patients opted for surgical treatment of both the cubital tunnel and Guyon’s canal. They were thoroughly informed about the results of their electromyographic and radiological examinations, which allowed them to make their own decisions. All patients chose to have simultaneous surgery on both areas. Out of these four patients, three showed ulnar nerve instability and underwent anterior transposition. The other two underwent simple decompression due to ulnar nerve swelling, with no other notable findings. The Guyon’s canal was accessed in all patients through the pisiform and hamate, releasing Zones I, II, and III. The conservative treatment method was used for patients with McGowan stage I, and typically lasted an average of 3 months. After ruling out any space-occupying lesions through radiological examination, we opted for conservative treatment. We evaluated whether to continue with this treatment based on symptom improvement, conducting follow-ups at 6-week intervals for a 3-month period. The conservative treatment primarily involved educating patients on lifestyle modifications and subsequently monitoring symptoms for progression. We advise against assuming provocative positions and movements, cautioning patients to avoid sleeping in a prone position, resting elbows on desks while using the phone, or participating in activities that excessively flex the elbow joint during daily routines. In severe cases, patients were advised to wear a night splint such as elbow brace. Additionally, they were instructed to perform ulnar nerve gliding exercises or similar nerve stretching exercises at least three times daily [9]. 

## Results

**Fig. 2:**
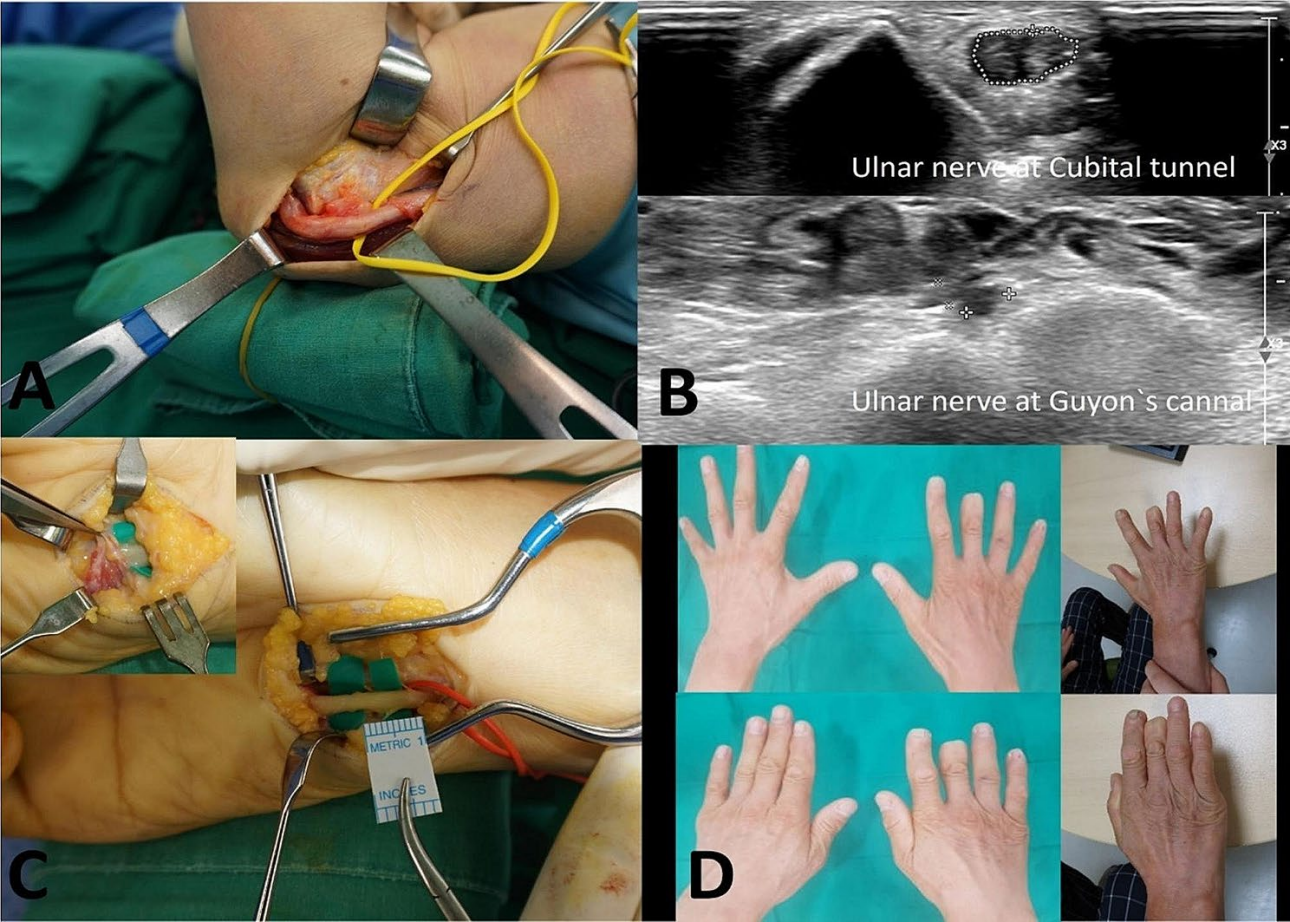
A sixty-one years old male came for right 4,5th finger tingling sensation and 1st webspace atrophy of right hand. Double crush syndrome was confirmed by EMG/NCS with Cubital tunnel syndrome and Guyon’s canal syndrome. (**A**) Intraoperative exploration, the ulnar nerve was compressed with swelling at the right elbow, with nerve dislocation upon flexion the elbow to 90 degrees. (**B**) The preoperative ultrasonography of right elbow view shows segmental swelling of ulnar nerve in cubital tunnel and ultrasonography of right wrist view shows a thicker deep branch of the ulnar nerve in Guyon’s canal. (**C**) Intraoperative exploration, the ulnar nerve was compressed with swelling exerted by the short palmaris brevis muscle at the right wrist. (**D**) On physical examination of last follow up, 1st web space atrophy was improved. (Mcgowan stage 0)

In all 4 patients who underwent surgical intervention, ulnar nerve compression and swelling were observed at the elbow joint. Consequently, ulnar nerve transposition or release procedures were performed. Surgical treatment was administered at the wrist in 4 patients. In the surgical findings, ulnar nerve swelling was confirmed at the medial and inferior cubital joints in all patients. In 3 cases, anterior transposition surgery was performed subsequent to the observation of ulnar nerve subluxation at 90 degrees of elbow flexion. No evidence of space-occupying lesions or abnormal muscle hypertrophy was observed in the elbow region. At the wrist level during surgical exploration, the deep motor branch of Zone II was compressed by a fibrous band at the pisohamate opening in 2 cases. Additionally, there was 1 case in which nerve swelling extended across Zone I, concomitant with compression exerted by the short palmaris brevis muscle (Fig. 2). One other case showed nerve compression attributed to ganglion connected to the pisohamate joint, which was excised during surgery.

After surgery, a noticeable improvement in clinical symptoms was observed. Upon final assessment, 2 of 4 patients achieved stage 0 on the McGowan scale, denoting complete alleviation of symptoms, and reported significant satisfaction. In the remaining 2 cases, McGowan scale progressed from stage IIB to IIA, with subjective satisfaction noted as moderate. Visual examination revealed mild web space atrophy in 2 patients. Two patients exhibited slight discomfort when performing fine motor tasks such as using chopsticks or nail clippers. When patient satisfaction was stratified into categories of excellent, good, fair, and poor, 2 patients were categorized as ‘excellent,’ while the remaining two were classified as ‘good.’ In the 3 cases where conservative treatment was administered instead of surgery, subjective satisfaction was ‘excellent’ in 1 case and ‘good’ in 2 cases.

All patients who received conservative treatment showed clinical improvement within the initial 3 months, and after maintaining conservative treatment for at least 6 months, the treatment was ended.

One patient (No. 3) who underwent surgical treatment had a cubital tunnel syndrome (Mc gowan stage IIb) on the opposite side (left) and underwent surgery at intervals of one month. As of 17 months, the condition was IIA. One patient (No.7) who received conservative treatment also had Cubital tunnel syndrome (Mc gowan stage IIa) on the opposite side (right), and there was no improvement in symptoms on both sides during conservative treatment, so right side underwent ulnar nerve simple decompression. As of 10 months, the left side was Mcgowan stage I, and the right side was Mcgowan stage I 3 months after surgery.

The mean follow-up period for patients who underwent surgery was 14.6 months. There were no specific complications or recurrence at the final follow-up. The mean follow-up period for patients who received conservative treatment was 10.3 months. Among all 8 cases, the preoperative average score for digital function was 0.88 (ranging from 0 to 2 points), while the postoperative average was 1.4 (ranging from 0 to 2 points), indicating overall improvement in outcomes.

## Discussion

The concept of DCS posits that a proximal lesion in a nerve, resulting from impaired axonal transport, renders the distal portion vulnerable as well [1]. In cases of DCS asymptomatic compression of the affected nerve is believed to reduce nutrient flow in both antegrade and retrograde directions, ultimately leading to axonal rupture [10]. This increases the vulnerability of the distal axon and gives rise to symptoms resembling those of compression syndrome [1, 11]. However, DCS is not solely a neurogenic disorder determined by anatomical location. It should also be regarded as a potential occurrence in various medical conditions such as diabetes, infection, hypothyroidism, and vitamin deficiency, which could result in multiple peripheral neuropathies. There is no definitive confirmatory test available. and Accurate diagnosis relies on thorough medical history-taking, physical examination, imaging studies, and EMG.

The key clinical observation for distinguishing between cubital tunnel syndrome(CuTS) and Guyon`s canal syndrome(GCS) is a sensory deficit in the ulnar dorsum of the hand. The ulnar nerve emits the dorsal cutaneous branch approximately 8 cm proximal to the pisiform bone, which innervates the dorsal-ulnar aspect of the hand. EMG is also conducted based on these physiological characteristics. A typical EMG performed on patients presenting with complaints of numbness in the 4th and 5th fingers and atrophy of the intrinsic muscles first assesses the conduction velocity at the cubital joint to ascertain the presence or absence of cubital tunnel syndrome. If no specific results are obtained here, additional testing is carried out at the wrist to confirm ulnar canal syndrome. If conduction velocity is slowed at the cubital joint, it is diagnosed as CuTS, and no further tests are conducted at the wrist. If there is suspicion of both conditions coexisting in a patient, ulnar nerve conduction velocity must be examined at both the elbow and wrist. However, given the rarity of concurrent CuTS and GCS cases, administering these tests to all patients can become a time-consuming process and may pose a challenge in terms of cost-effectiveness.

To date, there exists a controversy with DCS, wherein the detection of abnormal EMG findings in both regions may raise questions about true symptomatic neuropathy. In contrast, if neuropathy is suspected in both locations but surgical intervention is only conducted in one, ethical concerns may arise despite obtaining patient consent. This incongruity represents the primary limitation in validating DCS [12]. In a recent paper comparing two groups of DCS of the ulnar nerve including CuTS with GCS and isolated GCS, it was reported that patients with isolated GCS recovered their grip strength faster after surgery, but the final outcome was similar [6]. . In addition, a comparison was made between the group that had surgery on both sides in patients with CuTS accompanied by cervical radiculopathy (the group that first had surgery for CuTS and the group that first had Anterior Cervical Discectomy and Fusion (ACDF)) and the group that was diagnosed with only cubital tunnel syndrome and had surgery [13]. There was no difference in the outcome between DCS and isolated cuts. However, poor outcomes within the DCS group were primarily linked to symptom duration exceeding 1 year, a history of multiple neuropathy or radiculopathy, and previous ACDF surgery performed before ulnar nerve release. These results suggest that DCS indeed has clinical significance [14]. 

To date, cases of double compression of the ulnar nerve including CuTS with GCS are exceedingly rare [6, 15, 16]. Physician who perform neurosurgery must consider this and definitely require improved electromyography [10]. In the management of patients suspected of double crush syndrome, it is crucial to emphasize thorough patient explanation, given the current lack of clear guidelines. Subsequently, relatively invasive treatments, including surgery, should be initiated preferentially at sites exhibiting greater clinical suspicion or offering easier surgical access. In this study, we provided comprehensive explanations of the results of preoperative EMG and ultrasound imaging tests to patients, aiming to enhance their understanding of double crush syndrome. Furthermore, we underscored the potential discordance between diagnostic findings and neurological symptoms. Subsequent to thorough consultation, surgical intervention strategies were determined, contemplating whether to address both sites simultaneously or to adopt a phased approach. Finally, all 4 patients who underwent concurrent surgery at the cubital and guyon cannal expressed a preference for simultaneous intervention.

As a limitation of this study, it was not possible to generalize the characteristics of the patients. This is because it cannot be assumed that they all have the same pathophysiology in the same area. In addition, as a retrospective study with a small number of patients, it simply lists the results of treatment, and it was difficult to compare the effects of surgery.

## Conclusion

When assessing patients with CuTS, it’s crucial to consider an inching test during a nerve conduction study at elbow and wrist. If double crush syndrome is suspected but symptoms are mild, they may respond to conservative treatment similar to a single neuropathy. Subsequently, relatively invasive treatments, including surgery, should be initiated preferentially at sites exhibiting greater clinical suspicion or offering easier surgical access. This approach aims to limit ethical concerns.

## Ethical declarations

**Table 1:** Case series for double crush syndrome

Case	Sex/Age	Diagnosis	Involved Side	Symptom onset (month)	Pre-operative				Post-treatment			Outcome		Operative Finding	
					Hand Function (0,1,2)^2)^	4,5th finger numbness	Muscle atrophy	McGowan	Hand Function (0,1,2)	4,5th finger numbness	Muscle atrophy	McGowan	Satisfaction (0,1,2,3)^3)^	Cubital	Other site
1	M/61	GCS + CuTs^1)^	Left	3	1	Yes	+	IIB	2	No	maintained	IIA	2	Nerve swelling, hypoeremic change Hypertrophic synovium	Fibrous band at Guyon cannal(Zone II)
2	M/47	GCS + CuTs	Left	5	0 1	No	+	IIB	2	No	Abscent	0	3	Nerve swelling	Ganglion at Guyon cannal
3	M/66	GCS + CuTs	Right	1	1 0	No	+	IIB	1	No	Improved	IIA	3	Nerve dislocation at elbow flexion 90’ Nerve swelling	Fibrous band at Guyon cannal(Zone II)
4	M/61	GCS + CuTs	Right	120	1	Yes	+	IIB	2	Yes	Improved	0	3	Nerve dislocation at elbow flexion 90’ Nerve swelling	Palmaris Brevis Fibrotic band at Guyon cannal(Zone II )
5	M/60	GCS + CuTs	Right	2	1	Yes	Abscent	I	1	No	Abscent	I	2	-	-
6	F/13	GCS + CuTs	Right	1	1	Yes	Abscent	IIA	1	No	Abscent	0	3	-	-
7	F/47	GCS + CuTs	Left	4	1	Yes	Abscent	I	1	No	Abscent	I	2	-	-

1) GCS : Guyon canal syndrome, CuTs : Cubital tunnel syndrome, CM : Cervical myelopathy

2) Performance score of hand function such as tying shoelaces, using chopsticks, and buttoning clothes, 2 : perform properly, 1 : perform with difficulty, 0 : unable to perform

3) 3: significant improved, 2: moderate improved, 1: no improvement, 0 : deterioration

## Data Availability

The datasets used and analyzed during the current study are not publicly available due to lack of participant consent to share their data but are available from the corresponding author upon reasonable request after ethical considerations are met.
