# Correction: Double entrapment neuropathy of the ulnar nerve at the elbow and the wrist: double crush syndrome?

**DOI:** 10.1186/s12891-024-07690-w

**Published:** 2024-08-01

**Authors:** Dong Hee Kim, Sung Jin Shin, Jun Yong Park, Sang Hyun Lee

**Affiliations:** 1 Departments of Orthopedic Surgery, Samsung Changwon Hospital, Sungkyunkwan University School of Medicine, Changwon, Republic of Korea; 2 Department of Orthopaedic Surgery, Medical Research Institute, Pusan National University Hospital, Pusan National University School of Medicine, 179, Gudeok-ro seo-gu, Busan, 49241 Republic of Korea

**Correction:** ***BMC Musculoskelet Disord*** **25, 463 (2024)**

10.1186/s12891-024-07574-z

Following publication of the original article [1], the authors corrected a mistake in the affiliation of the third author Jun Yong Park. It was stated, “Department of Orthopaedic Surgery, Medical Research Institute, Pusan National University Hospital, Pusan National University School of Medicine, 179, Gudeok-ro seo-gu, Busan 49241, Republic of Korea” instead of the correct affiliation, “Departments of Orthopedic Surgery, Samsung Changwon Hospital, Sungkyunkwan University School of Medicine, Changwon, Republic of Korea”.

The original article [1] has been corrected.
